# Risks of stroke, its subtypes and atrial fibrillation associated with glucagon-like peptide 1 receptor agonists versus sodium-glucose cotransporter 2 inhibitors: a real-world population-based cohort study in Hong Kong

**DOI:** 10.1186/s12933-023-01772-0

**Published:** 2023-02-24

**Authors:** David Tak Wai Lui, Eric Ho Man Tang, Tingting Wu, Ivan Chi Ho Au, Chi Ho Lee, Yu Cho Woo, Kathryn Choon Beng Tan, Carlos King Ho Wong

**Affiliations:** 1 Division of Endocrinology and Metabolism, Department of Medicine, School of Clinical Medicine, LKS Faculty of Medicine, The University of Hong Kong, Hong Kong SAR, China; 2 Department of Family Medicine and Primary Care, School of Clinical Medicine, LKS Faculty of Medicine, The University of Hong Kong, Hong Kong SAR, China; 3 Centre for Safe Medication Practice and Research, Department of Pharmacology and Pharmacy, LKS Faculty of Medicine, The University of Hong Kong, Hong Kong SAR, China; 4 Laboratory of Data Discovery for Health Limited (D24H), Hong Kong Science Park, New Territories, Hong Kong SAR, China

**Keywords:** Sodium-glucose cotransporter 2 inhibitors, Glucagon-like peptide-1 receptor agonists, Stroke, Ischemic stroke, Atrial fibrillation

## Abstract

**Background:**

There are limited data on head-to-head comparative risk of stroke between sodium-glucose cotransporter 2 inhibitors (SGLT2i) and glucagon-like peptide-1 receptor agonists (GLP-1RA). We compared risk of stroke with its subtypes and incident atrial fibrillation (AF) between them.

**Methods:**

A population-based, retrospective cohort of patients with type 2 diabetes between 2008 and 2020 were identified from the electronic health records of Hong Kong Hospital Authority. Patients who received SGLT2i or GLP-1RA were matched pairwise by propensity score. Risks of stroke and AF were evaluated by hazard ratios (HRs) from the Cox proportional hazard regression models.

**Results:**

A total of 5840 patients (2920 SGLT2i users; 2920 GLP-1RA users) were included (mean age 55.5 years, 56.1% men, mean HbA1c 8.9% and duration of diabetes 13.7 years). Upon median follow-up of 17 months, there were 111 (1.9%) events of stroke (SGLT2i: 62, 2.1%; GLP-1RA: 49 1.7%). SGLT2i users had comparable risk of all stroke as GLP-1RA users (HR 1.46, 95% CI 0.99–2.17, p = 0.058). SGLT2i users had higher risk of ischemic stroke (HR 1.53, 95% CI 1.01–2.33, p = 0.044) but similar risk of hemorrhagic stroke compared to GLP-1RA users. Although SGLT2i was associated with lower risk of incident AF (HR 0.43, 95% CI 0.23–0.79, p = 0.006), risk of cardioembolic stroke was similar.

**Conclusions:**

Our real-world study demonstrated that GLP-1RA use was associated with lower risk of ischemic stroke, despite the association between SGLT2i use and lower risk of incident AF. There was no significant difference in hemorrhagic stroke risk. GLP-1RA may be the preferred agent for patients with type 2 diabetes at risk of ischemic stroke.

**Supplementary Information:**

The online version contains supplementary material available at 10.1186/s12933-023-01772-0.

## Background

Both sodium-glucose cotransporter 2 inhibitors (SGLT2i) and glucagon-like peptide-1 receptor agonists (GLP-1RA) are newer classes of anti-diabetic agents which have shown cardiorenal benefits in landmark trials [1]. These agents have expanded the armamentarium in the management of type 2 diabetes [2], providing benefits beyond glycemic control. Head-to-head comparisons between SGLT2i and GLP-1RA may provide further insights in the selection of appropriate anti-diabetic agents, currently limited in the literature especially regarding stroke risk. A recent network meta-analysis of cardiovascular outcome trials involving SGLT2i and GLP-1RA revealed that GLP-1RA statistically significantly reduced risk of non-fatal stroke by 16% compared with placebo, while GLP-1RA had a 10% lower risk of non-fatal stroke compared with SGLT2i but not reaching statistical significance [3]. Nonetheless, this network meta-analysis represents an indirect comparison of the cardiovascular outcome trials which requires cautions in data interpretation.

Few studies have evaluated the risks of other subtypes of stroke among SGLT2i and GLP-1RA users. Two meta-analyses of large randomized controlled trials of SGLT2i suggested that SGLT2i use was associated with reduced risk of hemorrhagic stroke but neutral on ischemic stroke compared with placebo [4, 5]. Regarding GLP-1RA, exploratory analysis of REWIND trial suggested that dulaglutide was associated with lower risk of ischemic stroke but neutral on hemorrhagic stroke compared with placebo [6]. Furthermore, SGLT2i was shown to be associated with lower risk of incident atrial fibrillation (AF) [7], but not for GLP-1RA [8]. Whether this may imply differential benefits on the risk of cardioembolic stroke between SGLT2i and GLP1-RA users remains to be elucidated.

We carried out this real-world population-based study of Chinese patients with type 2 diabetes who were started on SGLT2i or GLP-1RA to compare their risks of stroke with its subtypes and incident AF.

## Methods

### Study design and data source

This retrospective population-wide cohort study utilized electronic medical records from the Hong Kong Hospital Authority database. The Hospital Authority is the statutory body that provides public healthcare services in outpatient clinics and all public hospitals in Hong Kong, covering 90% of all secondary and tertiary care in the territory [9]. The service is available to all Hong Kong residents (> 7.3 million) [10]. Electronic medical records of patients include demographics, registered death records, laboratory tests, disease diagnoses, procedures, medication dispensing records, hospital admissions, outpatient and emergency department visits. Data from this database have been commonly used in diabetes-related research [10, 11]. Patients with type 2 diabetes managed in the Hospital Authority public health clinics receive regular diabetic complication screening. During each diabetic complication screening session, patients are assessed clinically and have laboratory investigations to determine their control of diabetes, its related cardiovascular risk factors and the presence of diabetic complications [10, 12]. Comorbidities are also recorded by clinicians as diagnosis codes of International Classification of Primary Care, second edition (ICPC-2) or the International Classification of Diseases, Ninth Revision, Clinical Modification (ICD-9-CM) when patients attend any clinic visits or are hospitalized. The use of medications refers to the dispensing records under the Hospital Authority when patients attend any clinic visits or are hospitalized.

### Subject’s inclusion and exclusion criteria

**Fig. 1:**
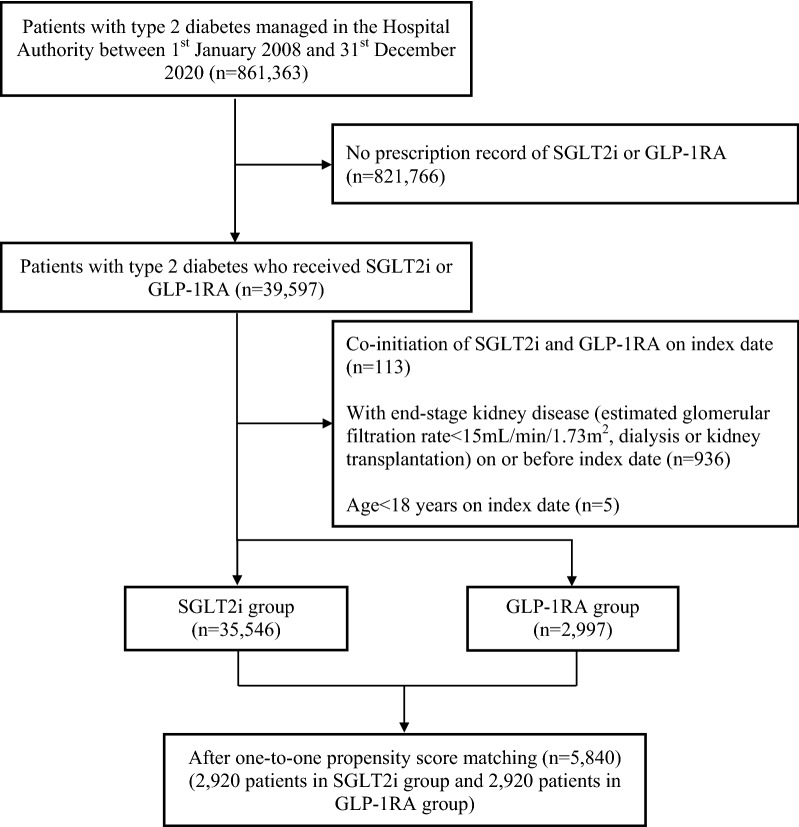
Flowchart of subject inclusion. The index date was defined as the first date of receiving SGLT2i or GLP-1RA. SGLT2i, sodium–glucose cotransporter-2 inhibitors; GLP-1RA, glucagon-like peptide-1 receptor agonist

We included patients with type 2 diabetes managed in all outpatient clinics and in inpatient settings in all hospitals under the Hospital Authority between 1st January 2008 and 31st December 2020 (Fig. 1). The starting date was set on 1st January 2008 because the first prescription of GLP-1RA was in 2008 and that for SGLT2i was in 2015, respectively. This ensured capturing all patients with type 2 diabetes newly initiated on these two classes of anti-diabetic agents.

The diagnosis of type 2 diabetes was defined by the ICPC-2 codes ‘T90’, the ICD-9-CM codes ‘250.x0’ and ‘250.x2’, or dispensing record of oral anti-diabetes medications.

The exposure of interest was the use of SGLT2i versus GLP-1RA. Patients with at least one dispensing record of SGLT2i or GLP-1RA were included. They were classified into either SGLT2i or GLP-1RA group. Patients who had ever received SGLT2i and GLP-1RA were assigned to the group of the novel anti-diabetic medication that were initiated earlier. In this study, active-comparator new-user design was adopted, and hence, no placebo group was included. The index date of each patient was set at the date of SGLT2i or GLP-1RA initiation. Patients who were aged < 18 years, had end-stage kidney disease (estimated glomerular filtration rate [eGFR] < 15 mL/min/1.73m^2^, dialysis or kidney transplantation) on or before the index date, or had co-initiation of SGLT2i and GLP-1RA at index date were excluded from the study.

After applying the inclusion and exclusion criteria, a total of 38,543 eligible patients were identified and assigned to SGLT2i (n = 35,546) and GLP-1RA (n = 2997) groups.

### Outcome measures

The primary outcome was all events of stroke, consisting of hemorrhagic and ischemic stroke. Secondary outcomes included hemorrhagic stroke, ischemic stroke, incident AF, and cardioembolic stroke.

Hemorrhagic, ischemic stroke, and AF were identified by ICD-9-CM codes ‘430’ to ‘432.x’, ‘433.x’ to ‘438.x’, and ‘427.31’, respectively. Cardioembolic stroke was identified among ischemic stroke, with the help of the TOAST cardioembolic features described in the study by Guan et al. using electronic health database [13]. For patients who had history of stroke before the index date, incident stroke was identified if the relevant diagnosis codes were listed as the primary diagnosis of the hospitalization records.

### Definitions of variables

Variables for confounding adjustment included demographics, clinical parameters, comorbidities, and use of medications in the six months preceding the index date. These were included in view of their clinical relevance. Clinical parameters included hemoglobin A1c (HbA1c), fasting glucose, systolic and diastolic blood pressure, low-density lipoprotein cholesterol, total cholesterol to high-density lipoprotein cholesterol ratio, triglyceride, body mass index (BMI), eGFR (≥ 60 or < 60 mL/min/1.73m^2^), albuminuria (normal if urine albumin creatinine ratio [ACR] < 3.4 mg/mmol Cr; microalbuminuria if urine ACR ≥ 3.4 to < 34 mg/mmol Cr; and macroalbuminuria if urine ACR ≥ 34 mg/mmol Cr), and duration of diabetes. Comorbidities comprised congestive heart failure, hemorrhagic and ischemic stroke, chronic obstructive pulmonary disease, liver disease, severe hypoglycemia, AF, vascular disease (including coronary heart disease and peripheral vascular disease), diabetic retinopathy and diabetic neuropathy. Medication use included anti-diabetic, anti-hypertensive, and lipid-lowering agents, antiplatelets, and anticoagulants.

Complete data on demographics, comorbidity status, and prescription records of medications were extracted, while the completion rate of laboratory parameters ranged from 62.9 to 99.2%. The data completion rates of baseline covariates between treatment groups are presented in Additional file 1: Table S1.

### Statistical analyses

Regarding baseline characteristics of the patients, continuous variables were reported as mean with standard deviation (SD) while categorical variables were reported as number with percentage. Multiple imputation by chained equation was used to impute missing baseline covariates [14, 15]. Each missing datum was imputed using known or imputed variables by five times. Five complete datasets were generated and analyzed independently, and the analyzed results were pooled to an overall estimate by using the Rubin’s rule [16]. One-to-one propensity score matching was performed to compare patients who had initiated SGLT2i or GLP-1RA at clinical equipoise. A logistic regression model was fitted to estimate the propensity score of patients by using the baseline covariates. Patients in SGLT2i and GLP-1RA groups were pairwise matched using the propensity score with a caliper of 0.001. An absolute standardized mean difference (ASMD) ≤ 0.1 of baseline covariate indicated the covariate balance between the two groups [17].

Each included patient had complete follow-up and was observed from the index date until any of the following: (i) the occurrence of events; (ii) death; (iii) addition or switching of treatments to another study exposures (e.g., patients who were initiated on SGLT2i first and switched to GLP-1RA were censored at the start date of GLP-1RA, and vice versa); or (iv) the date of the end of study (i.e., 31st December 2020), whichever was earlier. Cox proportion hazard regression models were constructed to estimate hazard ratio (HR) and 95% confidence interval (CI) of each outcome between the two groups. In addition, incidence rate ratio (IRR) for each outcome was estimated by Poisson regression models.

Subgroup analyses were performed by stratification according to sex (male and female), age group (< 60 and ≥ 60 years), HbA1c (< 8 and ≥ 8%), history of stroke, history of AF, and duration of diabetes (< 10 and ≥ 10 years). Tests for interaction between the novel anti-diabetic medications and subgroups were conducted.

Sensitivity analyses were conducted to check whether consistent results were obtained with different analytic approaches: (i) analysis without censoring on switching treatments; (ii) inclusion of patients with follow-up duration ≥ 1 year; (iii) inclusion of patients with at least two dispensing records within 12 months; (iv) inclusion of only patients who were initiated on SGLT2i or GLP-1RA in or after 2015; (v) ‘as-treated’ analysis, censoring when patients stopped being dispensed the index treatment; (vi) analysis using regression adjustment to control for confounders instead of propensity score matching; and (vii) analysis without adjustment of covariates using either propensity score matching or regression adjustment.

All statistical analyses were performed using Stata version 14.0 (StataCorp, College Station, Texas). p values < 0.05 were considered statistically significant. The current study was reported according to the guideline of STrengthening the Reporting of OBservational studies in Epidemiology (STROBE).

## Results

After one-to-one propensity matching between SGLT2i and GLP-1RA groups, 5840 patients (2920 patients in each group) were finally included. Figure 1 shows the study flowchart. Table 1 summarizes the baseline characteristics of included patients. The mean age was 55.5 years, and 56.1% were male. The mean HbA1c and BMI were 8.9% and 30.2 kg/m^2^, respectively. The median duration of SGLT2i and GLP-1RA cumulative use were 15.4 and 10.1 months (p < 0.001), respectively.

**Table 1:** Baseline characteristics of patients after multiple imputation and one-to-one propensity score matching

Factors, mean (standard deviation) or % (n)	All patients (N = 5840)	SGLT2i (N = 2920)	GLP-1RA (N = 2920)	ASMD
Sex				0.010
Male	56.1% (3274)	55.8% (1630)	56.3% (1644)	
Female	43.9% (2566)	44.2% (1290)	43.7% (1276)	
Age, years	55.5 (12.5)	55.4 (12.4)	55.6 (12.6)	0.017
*Clinical and laboratory parameters*				
Hemoglobin A1c, %	8.9 (1.7)	8.9 (1.7)	8.9 (1.7)	0.029
Fasting glucose, mmol/L	9.5 (3.6)	9.5 (3.6)	9.5 (3.6)	0.010
Systolic blood pressure, mmHg	137.4 (23.0)	137.1 (22.1)	137.7 (22.1)	0.028
Diastolic blood pressure, mmHg	79.0 (14.5)	78.9 (14.9)	79.2 (14.1)	0.017
Low-density lipoprotein cholesterol, mmol/L	2.3 (0.8)	2.3 (0.8)	2.2 (0.8)	0.009
Total cholesterol to high-density lipoprotein cholesterol ratio	4.0 (1.3)	4.0 (1.4)	4.0 (1.2)	0.019
Triglyceride, mmol/L	2.0 (1.4)	2.0 (1.5)	2.0 (1.4)	0.046
Body mass index, kg/m^2^	30.2 (6.6)	29.9 (6.7)	30.4 (6.5)	0.087
Estimated glomerular filtration rate				0.020
≥ 60 mL/min/1.73m^2^	72.2% (4217)	72.6% (2121)	71.8% (2095)	
< 60 mL/min/1.73m^2^	27.8% (1623)	27.4% (799)	28.2% (825)	
Status of microalbuminuria/macroalbuminuria				0.016
Normal	44.4% (2591)	44.2% (1290)	44.6% (1,301)	
Microalbuminuria	34.6% (2019)	35.0% (1021)	34.2% (999)	
Macroalbuminuria	21.1% (1229)	20.9% (609)	21.2% (620)	
Duration of diabetes, year	13.7 (8.8)	13.7 (8.7)	13.8 (9.0)	0.010
*Comorbidities*				
Atrial fibrillation	2.2% (131)	2.1% (62)	2.4% (69)	0.016
Congestive heart failure	4.4% (259)	4.3% (127)	4.5% (132)	0.008
Stroke	6.0% (351)	5.7% (166)	6.3% (185)	0.027
Hemorrhagic stroke	1.0% (58)	0.9% (25)	1.1% (33)	0.028
Ischemic stroke	5.4% (317)	5.2% (151)	5.7% (166)	0.023
Chronic obstructive pulmonary disease	3.3% (191)	3.4% (100)	3.1% (91)	0.017
Liver disease	0.9% (52)	0.9% (27)	0.9% (25)	0.007
Severe hypoglycemia	4.9% (288)	4.9% (143)	5.0% (145)	0.003
Vascular disease	13.4% (783)	13.2% (384)	13.7% (399)	0.015
Diabetic retinopathy	15.2% (890)	15.3% (448)	15.1% (442)	0.006
Diabetic neuropathy	3.7% (216)	3.8% (110)	3.6% (106)	0.007
*Baseline medications*				
Anti-diabetic medications				
Insulin	71.8% (4192)	73.0% (2131)	70.6% (2061)	0.053
Metformin	84.2% (4918)	84.7% (2474)	83.7% (2444)	0.028
Sulfonylurea	43.9% (2564)	43.5% (1271)	44.3% (1293)	0.015
Thiazolidinedione	23.5% (1372)	24.0% (701)	23.0% (671)	0.024
Dipeptidyl peptidase 4 inhibitors	37.7% (2201)	37.8% (1103)	37.6% (1098)	0.004
Alpha-glucosidase inhibitors	1.7% (98)	1.4% (42)	1.9% (56)	0.037
Anti-hypertensive medications				
Angiotensin-converting enzyme inhibitors/angiotensin receptor blockers	74.9% (4372)	74.5% (2174)	75.3% (2198)	0.019
Beta blockers	39.3% (2297)	39.0% (1139)	39.7% (1158)	0.013
Calcium channel blockers	57.3% (3348)	57.4% (1675)	57.3% (1673)	0.001
Diuretics	21.1% (1233)	20.9% (610)	21.3% (623)	0.011
Other anti-hypertensive medications	10.1% (588)	9.8% (286)	10.3% (302)	0.018
Lipid-lowering agents	77.9% (4550)	77.7% (2270)	78.1% (2280)	0.008
Antiplatelets	28.0% (1636)	27.4% (801)	28.6% (835)	0.026
Anticoagulants	1.6% (93)	1.5% (43)	1.7% (50)	0.019
*Type of SGLT2i*				
Canagliflozin	NA	0.3% (9)	NA	NA
Dapagliflozin		36.6% (1068)		
Empagliflozin		63.3% (1847)		
*Type of GLP-1RA*				
Dulaglutide	NA	NA	30.5% (892)	NA
Exenatide			18.9% (553)	
Liraglutide			46.5% (1359)	
Lixisenatide			4.0% (117)	

SGLT2i, Sodium-glucose cotransporter-2 inhibitors; GLP-1RA, Glucagon-like peptide-1 receptor agonist; ASMD, Absolute standardized mean difference; NA, Not applicable

### Primary outcomes

The incidence and risk of stroke of patients used SGLT2i or GLP-1RA are shown in Table 2. With a median follow-up of 17 months (interquartile range: 8–34), the incidence rate of composite stroke in SGLT2i and GLP-1RA groups were 1.10 and 0.80 cases/100 person-years, respectively. Regarding events of all stroke, no significant difference in the risks was observed between SGLT2i and GLP-1RA groups (HR 1.46, 95% CI 0.99–2.17, p = 0.058; IRR 1.38, 95% CI 0.95–2.01, p = 0.089).

**Table 2:** Incidence and risk of stroke and atrial fibrillation between SGLT2i and GLP-1RA groups after one-to-one propensity score matching

	Number of case (%)	Incidence rate per 100 person-years (95% CI)	Person-years	Median follow-up month (IQR)	Incidence rate ratio (95% CI)	P-value	Hazard ratio (95% CI)	P-value
*All stroke*								
All patients	111 (1.9%)	0.94 (0.78,1.14)	11,771	17 (8–34)				
SGLT2i	62 (2.1%)	1.10 (0.86,1.41)	5621	19 (8–35)	1.38 (0.95,2.01)	0.089	1.46 (0.99,2.17)	0.058
GLP-1RA	49 (1.7%)	0.80 (0.60,1.05)	6150	17 (8–34)	1 (Reference)		1 (Reference)	
*Hemorrhagic stroke*								
All patients	23 (0.4%)	0.19 (0.13,0.29)	11,918	17 (8–34)				
SGLT2i	11 (0.4%)	0.19 (0.11,0.35)	5683	19 (9–35)	1.01 (0.44,2.28)	0.989	1.29 (0.53,3.14)	0.582
GLP-1RA	12 (0.4%)	0.19 (0.11,0.34)	6236	16 (7–32)	1 (Reference)		1 (Reference)	
*Ischemic stroke*								
All patients	98 (1.7%)	0.83 (0.68,1.01)	11,780	17 (8–34)				
SGLT2i	57 (2.0%)	1.01 (0.78,1.31)	5628	19 (8–35)	1.52 (1.02,2.27)	0.041*	1.53 (1.01,2.33)	0.044*
GLP-1RA	41 (1.4%)	0.67 (0.49,0.91)	6152	16 (7–32)	1 (Reference)		1 (Reference)	
*Cardioembolic stroke*								
All patients	25 (0.4%)	0.21 (0.14,0.31)	11,906	17 (8–34)				
SGLT2i	14 (0.5%)	0.25 (0.15,0.42)	5676	19 (9–35)	1.40 (0.63,3.08)	0.407	1.40 (0.62,3.17)	0.414
GLP-1RA	11 (0.4%)	0.18 (0.10,0.32)	6230	16 (7–32)	1 (Reference)		1 (Reference)	
*Incident atrial fibrillation*								
All patients	50 (0.9%)	0.43 (0.33,0.57)	11,648	17 (8–34)				
SGLT2i	15 (0.5%)	0.27 (0.16,0.45)	5574	19 (9–35)	0.47 (0.26,0.86)	0.014*	0.43 (0.23,0.79)	0.006*
GLP-1RA	35 (1.2%)	0.58 (0.41,0.80)	6074	16 (7–32)	1 (Reference)		1 (Reference)	

The baseline covariates included in the logistic regression model for propensity score matching were sex, age, hemoglobin A1c, fasting glucose, systolic and diastolic blood pressure, low-density lipoprotein cholesterol, total cholesterol to high-density lipoprotein cholesterol ratio, triglyceride, body mass index, estimated glomerular filtration rate, albuminuria status, duration of diabetes, history of congestive heart failure, hemorrhagic and ischemic stroke, chronic obstructive pulmonary disease, liver disease, severe hypoglycemia, atrial fibrillation, vascular diseases, diabetic retinopathy and diabetic neuropathy, and use of anti-diabetic, anti-hypertensive, lipid-lowering agents, antiplatelets, and anticoagulants

SGLT2i, Sodium-glucose cotransporter-2 inhibitors; GLP-1RA, Glucagon-like peptide-1 receptor agonist; CI, Confidence interval; IQR, Interquartile range

*Significant at 0.05 level by Poisson regression model or Cox proportional hazard regression model, as appropriate

### Secondary outcomes

When analyzed according to subtypes of stroke, we observed a statistically significantly higher risk of ischemic stroke among SGLT2i users compared with GLP-1RA users (HR 1.53, 95% CI 1.01–2.33, p = 0.044; IRR 1.52, 95% CI 1.02–2.27, p = 0.041), whereas the risk of hemorrhagic stroke (HR 1.29, 95% CI 0.53–3.14, p = 0.582; IRR 1.01, 95% CI 0.44–2.28, p = 0.989) and cardioembolic stroke (HR 1.40, 95% CI 0.62–3.17, p = 0.414; IRR 1.40, 95% CI 0.63–3.08, p = 0.407) was comparable between groups. The incidence rate of AF in the SGLT2i group and GLP-1RA were 0.27 and 0.58 cases/100 person-years respectively. The risk of incident AF was statistically significantly lower among SGLT2i users (HR 0.43, 95% CI 0.23–0.79, p = 0.006; IRR 0.47, 95% CI 0.26–0.86, p = 0.014).

### Subgroup analyses

We performed subgroup analyses on the primary outcome and secondary outcome of ischemic stroke, with results shown in Additional file 1: Tables S2 and S3 respectively. Regarding all stroke, no significant interaction was observed between subgroups by age, sex, glycemic control and history of AF. However, patients with history of stroke appeared to benefit more from GLP-1RA use (p for interaction = 0.043) than those without history of stroke. Moreover, patients with shorter duration of diabetes (< 10 years) also appeared to benefit more from GLP-1RA use than those with longer duration of diabetes (p for interaction = 0.017). When focusing on ischemic stroke, we observed no interaction across subgroups by age, sex, glycemic control, history of stroke and history of AF. Again, patients with shorter duration of diabetes (< 10 years) appeared to benefit more from GLP-1RA than those with longer duration of diabetes (p for interaction = 0.009).

### Sensitivity analyses

Main results were consistent to those obtained from analyses using different analytic approaches, namely: (i) analysis without censoring on switching treatments; (ii) inclusion of patients with follow-up duration ≥ 1 year; (iii) inclusion of patients with at least two dispensing records within 12 months; (iv) inclusion of only patients who were initiated on SGLT2i or GLP-1RA in or after 2015; (v) ‘as-treated’ analysis, censoring when patients stopped being dispensed the index treatment; (vi) analysis using regression adjustment to control for confounders instead of propensity score matching; and (vii) analysis without adjustment of covariates using either propensity score matching or regression adjustments (Additional file 1: Table S4).

## Discussion

Our real-world population-based propensity-score matched cohort study of Chinese patients with type 2 diabetes was among the few head-to-head comparisons dedicated to risks of stroke between SGLT2i and GLP-1RA use. Upon a median follow-up of 17 months, there was no significant difference in the risk of all stroke between SGLT2i and GLP-1RA users. Regarding the subtype of stroke, GLP-1RA use was associated with lower risk of ischemic stroke compared with SGLT2i use. On the other hand, there was no significant difference in the risk of hemorrhagic stroke between SGLT2i and GLP-1RA. SGLT2i use was associated with lower risk of incident AF compared with GLP-1RA use. Our results suggested that, among the newer classes of anti-diabetic agents, GLP-1RA may be preferred for patients with type 2 diabetes at risk of ischemic stroke.

Head-to-head comparison of the risk of stroke between SGLT2i and GLP-1RA users was performed only in a few real-world studies. A recent summary of real-world studies suggested that SGLT2i and GLP-1RA initiators were at a similar risk of stroke (HR 1.01, 95% CI 0.93–1.10) [18]. However, these had a relatively short follow-up time of < 1 year. Patorno et al. [19] evaluated using United States claims database and showed no significant difference in risk of stroke (composite of ischemic and hemorrhagic) between the two groups of users. There was also no heterogeneity when analyzed according to history of cardiovascular diseases. The same group also reported comparable risks of stroke between SGLT2i and GLP-1RA users among older individuals with type 2 diabetes [20]. An earlier study of a smaller scale using US claims database showed similar risks of ischemic stroke between the two groups [21]. DeRemer et al. reported that addition of SGLT2i or GLP-1RA to metformin resulted in similar risks of stroke (subtype not specified) [22]. Studies from Danish nationwide database [23] and Italian electronic health database [24] also demonstrated similar stroke risk (subtype not specified) respectively. Nonetheless, important laboratory results regarding glycemic control and renal function were missing in some of the datasets, which may modify the comparative risk of stroke. Lugner et al. evaluated using Swedish national database with more complete laboratory parameters showed that SGLT2i use might be inferior to GLP-1RA regarding risks of stroke (composite of ischemic and hemorrhagic stroke) (HR 1.44, 95% CI 0.99–2.08, p = 0.056) [25]. Lately, two real-world studies with longer follow-up time of around 1.5 years demonstrated higher risk of ischemic stroke with SGLT2i use compared with GLP-1RA use in the Swedish database (HR for SGLT2i use *vs* GLP-1RA = 1.71, 95% CI 1.14–2.59) [26] and the Scandinavian nationwide database (HR 1.14, 95% CI 1.03–1.26) [27]. With a median follow-up of 17 months, we showed that there might be benefit in all stroke with GLP-1RA use compared with SGLT2i in the context of secondary prevention as revealed in the subgroup analysis where GLP-1RA use was associated with statistically significant lower risk of all stroke compared with SGLT2i use in patients with history of stroke. Regarding the specific subtype of stroke, GLP-1RA use showed superiority over SGLT2i in terms of ischemic stroke. Interestingly, subgroup analysis revealed that the superiority with GLP-1RA use in stroke risk (all stroke and ischemic stroke) was more prominent in patients with shorter duration of diabetes, which may suggest the benefit in early initiation of GLP-1RA to derive the maximal protection from the risk of stroke. Taken together, our results may support the preferential use of GLP-1RA in patients with type 2 diabetes and history of stroke.

In contrast to ischemic stroke, there was less extensive information on hemorrhagic stroke risk for the novel anti-diabetic agents. Some evidence showed the benefits of SGLT2i on hemorrhagic stroke in comparison to placebo. In two meta-analyses [4, 5], SGLT2i use was associated with a 50% reduction in the risk of hemorrhagic stroke, postulated to be related blood pressure lowering effect of SGLT2i, which has a more prominent benefit in hemorrhagic stroke [28]. With regard to the potential impact of GLP-1RA on hemorrhagic stroke, pre-clinical studies showed the potential role of GLP-1/GLP-1R axis in reducing hemorrhagic transformation after ischemic stroke [29]. However, post-hoc analyses of randomized controlled trials of semaglutide [30] and dulaglutide [6] did not demonstrate benefit with GLP-1RA use in hemorrhagic stroke compared with placebo. Our current head-to-head real-world study showed that SGLT2i and GLP-1RA were comparable in terms of the risk of hemorrhagic stroke.

We observed that SGLT2i use was associated with a lower risk of incident AF compared with GLP-1RA use, consistent with the existing literature [31]. SGLT2i use was shown to be associated with lower risk of incident AF compared with placebo [7]. In contrast, results from both meta-analyses of randomized controlled trials and observational studies showed no significant benefits with GLP-1RA use on reducing incident AF compared with placebo [8]. As AF is closely linked to cardioembolic stroke [32], whether this benefit in the reduction of incident AF translates into reduction of cardioembolic stroke has been evaluated as well. A meta-analysis of 6 trials reporting cardioembolic stroke as an outcome showed that SGLT2i use was associated with a lower risk of cardioembolic stroke compared with placebo [33]. Among GLP-1RA, this has been evaluated in the post-hoc analysis of randomized controlled trials of semaglutide, revealing only 11 events of cardioembolic stroke among 6,480 participants, not affected by semaglutide use [30]. Our current study did not reveal difference in the events of cardioembolic stroke between SGLT2i and GLP-1RA users upon a median follow-up of 17 months, which might be due to the relatively small number of events. Longer follow-up with larger cohorts may be necessary to clarify the comparative risk of cardioembolic stroke between SGLT2i and GLP-1RA users.

Our results should be interpreted bearing certain limitations. First, the duration of follow-up was relatively short, such that the number of events was relatively small for subgroup analyses. Second, all subjects in our cohort were Chinese, and our study did not cover all agents within SGLT2i and GLP-1RA classes (predominantly empagliflozin and dapagliflozin in the SGLT2i group, and liraglutide and dulaglutide in the GLP-1RA group). These limit the generalizability of results. Third, like all large-scale pharmacovigilance studies using electronic medical record databases, drug adherence could not be ascertained. Fourth, lifestyle factors related to cardiovascular diseases, such as dietary habits and physical activity, were not available in the database. Fifth, the current study design could not directly examine the statistical relationship between the incidence of stroke and the incidence of AF [32]. Last but not least, despite our attempts in balancing a range of patient characteristics by propensity score matching, in common with all epidemiological studies, retrospective database analysis cannot exclude residual confounders and infer causation.

## Conclusions

This population-based propensity-score matched observational cohort study of Chinese patients with type 2 diabetes demonstrated that GLP-1RA use was associated with a lower risk of ischemic stroke compared with SGLT2i use, whereas SGLT2i use was associated with a lower risk of incident AF compared with GLP-1RA use. On the other hand, there was no significant difference observed in the risk of hemorrhagic stroke between SGLT2i and GLP-1RA use. Our results suggested that GLP-1RA may be the preferred agent over SGLT2i for patients with type 2 diabetes at risk of ischemic stroke.

## Supplementary Information

**Additional file 1: Table S1.** Data complete rate of baseline covariates. **Table S2.** Subgroup analysis of risk of all stroke between SGLT2i and GLP-1RA groups. **Table S3. **Subgroup analysis of risk of ischemic stroke between SGLT2i and GLP-1RA groups. **Table S4.** Sensitivity analyses of stroke risk between SGLT2i and GLP-1RA groups.

## Data Availability

The data that support the findings of this study were provided by the Hong Kong Hospital Authority. Restrictions apply to the availability of these data, which were used under license for this study.

## Abbreviations

ACR: Albumin creatinine ratio
AF: Atrial fibrillation
ASMD: Absolute standardized mean difference
BMI: Body mass index
CI: Confidence interval
eGFR: Estimated glomerular filtration rate
GLP-1RA: Glucagon-like peptide-1 receptor agonists
HbA1c: Hemoglobin A1c
HR: Hazard ratio
ICD-9-CM: International Classification of Diseases, Ninth Revision, Clinical Modification
ICPC-2: International Classification of Primary Care, second edition
SD: Standard deviation
SGLT2i: Sodium-glucose cotransporter 2 inhibitors

## References

[1] Brown E, Heerspink HJL, Cuthbertson DJ, Wilding JPH (2021). SGLT2 inhibitors and GLP-1 receptor agonists: established and emerging indications. Lancet.

[2] Draznin B, Aroda VR, Bakris G, Benson G, Brown FM, American Diabetes Association Professional Practice Committee (2022). Pharmacologic approaches to glycemic treatment: standards of medical care in diabetes—2022. Diabetes Care..

[3] Giugliano D, Longo M, Signoriello S, Maiorino MI, Solerte B, Chiodini P (2022). The effect of DPP-4 inhibitors, GLP-1 receptor agonists and SGLT-2 inhibitors on cardiorenal outcomes: a network meta-analysis of 23 CVOTs. Cardiovasc Diabetol.

[4] Zhou Z, Jardine MJ, Li Q, Neuen BL, Cannon CP, de Zeeuw D (2021). Effect of SGLT2 inhibitors on stroke and atrial fibrillation in diabetic kidney disease: results from the CREDENCE trial and meta-analysis. Stroke.

[5] Tsai WH, Chuang SM, Liu SC, Lee CC, Chien MN, Leung CH (2021). Effects of SGLT2 inhibitors on stroke and its subtypes in patients with type 2 diabetes: a systematic review and meta-analysis. Sci Rep.

[6] Gerstein HC, Hart R, Colhoun HM, Diaz R, Lakshmanan M, Botros FT (2020). The effect of dulaglutide on stroke: an exploratory analysis of the REWIND trial. Lancet Diabetes Endocrinol.

[7] Zheng RJ, Wang Y, Tang JN, Duan JY, Yuan MY, Zhang JY (2022). Association of SGLT2 inhibitors with risk of atrial fibrillation and stroke in patients with and without type 2 diabetes: a systemic review and meta-analysis of randomized controlled trials. J Cardiovasc Pharmacol.

[8] Scheen AJ (2022). Antidiabetic agents and risk of atrial fibrillation/flutter: a comparative critical analysis with a focus on differences between SGLT2 inhibitors and GLP-1 receptor agonists. Diabetes Metab.

[9] Lui DTW, Lee CH, Chow WS, Fong CHY, Woo YC, Lam KSL (2020). A territory-wide study on the impact of COVID-19 on diabetes-related acute care. J Diabetes Investig.

[10] Lui DTW, Au ICH, Tang EHM, Cheung CL, Lee CH, Woo YC (2022). Kidney outcomes associated with sodium-glucose cotransporter 2 inhibitors versus glucagon-like peptide 1 receptor agonists: a real-world population-based analysis. EClinicalMedicine.

[11] Wong CKH, Lau KTK, Tang EHM, Lee CH, Lee CYY, Woo YC (2022). Cardiovascular benefits of SGLT2&nbsp;inhibitors in type 2 diabetes, interaction with metformin and role of erythrocytosis: a self-controlled case series study. Cardiovasc Diabetol.

[12] Tang EHM, Mak IL, Tse ETY, Wan EYF, Yu EYT, Chen JY (2022). Ten-year effectiveness of the multidisciplinary risk assessment and management programme-diabetes mellitus (RAMP-DM) on macrovascular and microvascular complications and all-cause mortality: a population-based cohort study. Diabetes Care.

[13] Guan W, Ko D, Khurshid S, Trisini Lipsanopoulos AT, Ashburner JM, Harrington LX (2021). Automated electronic phenotyping of cardioembolic stroke. Stroke.

[14] Raghunathan TW, Lepkowksi JM, Van Hoewyk J, Solenbeger P (2001). A multivariate technique for multiply imputing missing values using a sequence of regression models. Surv Methodol.

[15] van Buuren S (2007). Multiple imputation of discrete and continuous data by fully conditional specification. Stat Methods Med Res.

[16] Rubin DB (2004). Multiple imputation for nonresponse in surveys.

[17] Austin PC (2009). Balance diagnostics for comparing the distribution of baseline covariates between treatment groups in propensity-score matched samples. Stat Med.

[18] Caruso I, Cignarelli A, Sorice GP, Natalicchio A, Perrini S, Laviola L (2022). Cardiovascular and renal effectiveness of GLP-1 receptor agonists vs other glucose-lowering drugs in type 2 diabetes: a systematic review and meta-analysis of real-world studies. Metabolites..

[19] Patorno E, Htoo PT, Glynn RJ, Schneeweiss S, Wexler DJ, Pawar A (2021). Sodium–glucose cotransporter-2 inhibitors versus glucagon-like peptide-1 receptor agonists and the risk for cardiovascular outcomes in routine care patients with diabetes across categories of cardiovascular disease. Ann Intern Med.

[20] Patorno E, Pawar A, Bessette LG, Kim DH, Dave C, Glynn RJ (2021). Comparative effectiveness and safety of sodium-glucose cotransporter 2 inhibitors versus glucagon-like peptide 1 receptor agonists in older adults. Diabetes Care.

[21] Pineda ED, Liao IC, Godley PJ, Michel JB, Rascati KL (2020). Cardiovascular outcomes among patients with type 2 diabetes newly initiated on sodium–glucose cotransporter-2 inhibitors, glucagon-like peptide-1 receptor agonists, and other antidiabetic medications. J Manag Care Spec Pharm.

[22] DeRemer CE, Vouri SM, Guo J, Donahoo WT, Winterstein AG, Shao H (2021). Comparing cardiovascular benefits between GLP-1 receptor agonists and SGLT2 inhibitors as an add-on to metformin among patients with type 2 diabetes: a retrospective cohort study. J Diabetes Complicat.

[23] Norgaard CH, Starkopf L, Gerds TA, Vestergaard P, Bonde AN, Fosbol E (2022). Cardiovascular outcomes with GLP-1 receptor agonists vs. SGLT-2 inhibitors in patients with type 2 diabetes. Eur Heart J Cardiovasc Pharmacother..

[24] Longato E, Di Camillo B, Sparacino G, Gubian L, Avogaro A, Fadini GP (2020). Cardiovascular outcomes of type 2 diabetic patients treated with SGLT-2 inhibitors versus GLP-1 receptor agonists in real-life. BMJ Open Diabetes Res Care..

[25] Lugner M, Sattar N, Miftaraj M, Ekelund J, Franzen S, Svensson AM (2021). Cardiorenal and other diabetes related outcomes with SGLT-2 inhibitors compared to GLP-1 receptor agonists in type 2 diabetes: nationwide observational study. Cardiovasc Diabetol.

[26] Fu EL, Clase CM, Janse RJ, Lindholm B, Dekker FW, Jardine MJ (2022). Comparative effectiveness of SGLT2i versus GLP1-RA on cardiovascular outcomes in routine clinical practice. Int J Cardiol.

[27] Ueda P, Wintzell V, Dahlqwist E, Eliasson B, Svensson AM, Franzen S (2022). The comparative cardiovascular and renal effectiveness of sodium-glucose co-transporter-2 inhibitors and glucagon-like peptide-1 receptor agonists: a Scandinavian cohort study. Diabetes Obes Metab.

[28] Xie X, Atkins E, Lv J, Bennett A, Neal B, Ninomiya T (2016). Effects of intensive blood pressure lowering on cardiovascular and renal outcomes: updated systematic review and meta-analysis. Lancet.

[29] Zhang L, Zhang W, Tian X (2021). The pleiotropic of GLP-1/GLP-1R axis in central nervous system diseases. Int J Neurosci..

[30] Strain WD, Frenkel O, James MA, Leiter LA, Rasmussen S, Rothwell PM (2022). Effects of semaglutide on stroke subtypes in type 2 diabetes: post hoc analysis of the randomized SUSTAIN 6 and PIONEER 6. Stroke.

[31] Zhuo M, D'Andrea E, Paik JM, Wexler DJ, Everett BM, Glynn RJ (2022). Association of sodium–glucose cotransporter-2 inhibitors with incident atrial fibrillation in older adults with type 2 diabetes. JAMA Netw Open.

[32] Pistoia F, Sacco S, Tiseo C, Degan D, Ornello R, Carolei A (2016). The epidemiology of atrial fibrillation and stroke. Cardiol Clin.

[33] Li HL, Lip GYH, Feng Q, Fei Y, Tse YK, Wu MZ (2021). Sodium-glucose cotransporter 2 inhibitors (SGLT2i) and cardiac arrhythmias: a systematic review and meta-analysis. Cardiovasc Diabetol.

