# Supplementary material for: Risks of stroke, its subtypes and atrial fibrillation associated with glucagon-like peptide 1 receptor agonists versus sodium-glucose cotransporter 2 inhibitors: a real-world population-based cohort study in Hong Kong
Source: Cardiovasc Diabetol. 2023 Feb 24;22:40. doi: 10.1186/s12933-023-01772-0 (PMC9960638; doi:10.1186/s12933-023-01772-0)
Supplement: Supplementary file 1 — Additional file 1: Table S1. Data complete rate of baseline covariates. Table S2. Subgroup analysis of risk of all stroke between SGLT2i and GLP-1RA groups. Table S3. Subgroup analysis of risk of ischemic stroke between SGLT2i and GLP-1RA groups. Table S4. Sensitivity analyses of stroke risk between SGLT2i and GLP-1RA groups. [file 12933_2023_1772_MOESM1_ESM.docx]

**Title:** Risks of stroke, its subtypes and atrial fibrillation associated with glucagon-like peptide 1 receptor agonists versus sodium-glucose cotransporter 2 inhibitors: A real-world population-based study in Hong Kong

**Additional file 1**

**Table S1.** Data complete rate of baseline covariates

**Table S2.** Subgroup analysis of risk of all stroke between SGLT2i and GLP-1RA groups

**Table S3.** Subgroup analysis of risk of ischemic stroke between SGLT2i and GLP-1RA groups

**Table S4.** Sensitivity analyses of stroke risk between SGLT2i and GLP-1RA groups

**Table S1.** Data complete rate of baseline covariates

| Factors, % (n) | All patients  (N=38,543) | SGLT2i  (N=35,546) | GLP-1RA  (N=2,997) |
| --- | --- | --- | --- |
| Sex | 100.0% (38,543) | 100.0% (35,546) | 100.0% (2,997) |
| Age | 100.0% (38,543) | 100.0% (35,546) | 100.0% (2,997) |
|  |  |  |  |
| **Clinical and laboratory parameters** |  |  |  |
| Hemoglobin A1c | 98.3% (37,895) | 98.2% (34,923) | 99.2% (2,972) |
| Fasting glucose | 97.8% (37,676) | 97.6% (34,707) | 99.1% (2,969) |
| Systolic blood pressure | 76.7% (29,575) | 77.2% (27,456) | 70.7% (2,119) |
| Diastolic blood pressure | 76.7% (29,570) | 77.2% (27,452) | 70.7% (2,118) |
| Low-density lipoprotein cholesterol | 98.0% (37,756) | 97.9% (34,787) | 99.1% (2,969) |
| Total cholesterol to high-density lipoprotein cholesterol ratio | 98.1% (37,805) | 98.0% (34,836) | 99.1% (2,969) |
| Triglyceride | 98.2% (37,830) | 98.1% (34,861) | 99.1% (2,969) |
| Body mass index | 62.9% (24,229) | 63.3% (22,495) | 57.9% (1,734) |
| Estimated glomerular filtration rate | 99.2% (38,233) | 99.2% (35,249) | 99.6% (2,984) |
| Urine albumin to creatinine ratio | 86.6% (33,390) | 86.1% (30,619) | 92.5% (2,771) |
| Duration of diabetes | 100.0% (38,543) | 100.0% (35,546) | 100.0% (2,997) |
|  |  |  |  |
| **Comorbidities** |  |  |  |
| Atrial fibrillation | 100.0% (38,543) | 100.0% (35,546) | 100.0% (2,997) |
| Congestive heart failure | 100.0% (38,543) | 100.0% (35,546) | 100.0% (2,997) |
| Stroke | 100.0% (38,543) | 100.0% (35,546) | 100.0% (2,997) |
| Hemorrhagic stroke | 100.0% (38,543) | 100.0% (35,546) | 100.0% (2,997) |
| Ischemic stroke | 100.0% (38,543) | 100.0% (35,546) | 100.0% (2,997) |
| Chronic obstructive pulmonary disease | 100.0% (38,543) | 100.0% (35,546) | 100.0% (2,997) |
| Liver disease | 100.0% (38,543) | 100.0% (35,546) | 100.0% (2,997) |
| Severe hypoglycemia | 100.0% (38,543) | 100.0% (35,546) | 100.0% (2,997) |
| Vascular disease | 100.0% (38,543) | 100.0% (35,546) | 100.0% (2,997) |
| Diabetic retinopathy | 100.0% (38,543) | 100.0% (35,546) | 100.0% (2,997) |
| Diabetic neuropathy | 100.0% (38,543) | 100.0% (35,546) | 100.0% (2,997) |
|  |  |  |  |
| **Baseline medication** |  |  |  |
| Anti-diabetic medications |  |  |  |
| Insulin | 100.0% (38,543) | 100.0% (35,546) | 100.0% (2,997) |
| Metformin | 100.0% (38,543) | 100.0% (35,546) | 100.0% (2,997) |
| Sulfonylurea | 100.0% (38,543) | 100.0% (35,546) | 100.0% (2,997) |
| Thiazolidinedione | 100.0% (38,543) | 100.0% (35,546) | 100.0% (2,997) |
| Dipeptidyl peptidase 4 inhibitors | 100.0% (38,543) | 100.0% (35,546) | 100.0% (2,997) |
| Alpha-glucosidase inhibitors | 100.0% (38,543) | 100.0% (35,546) | 100.0% (2,997) |
| Anti-hypertensive medications |  |  |  |
| Angiotensin-converting enzyme inhibitors / angiotensin receptor blockers | 100.0% (38,543) | 100.0% (35,546) | 100.0% (2,997) |
| Beta blockers | 100.0% (38,543) | 100.0% (35,546) | 100.0% (2,997) |
| Calcium channel blockers | 100.0% (38,543) | 100.0% (35,546) | 100.0% (2,997) |
| Diuretics | 100.0% (38,543) | 100.0% (35,546) | 100.0% (2,997) |
| Other anti-hypertensive medications | 100.0% (38,543) | 100.0% (35,546) | 100.0% (2,997) |
| Lipid-lowering agents | 100.0% (38,543) | 100.0% (35,546) | 100.0% (2,997) |
| Antiplatelets | 100.0% (38,543) | 100.0% (35,546) | 100.0% (2,997) |
| Anticoagulants | 100.0% (38,543) | 100.0% (35,546) | 100.0% (2,997) |

SGLT2i = Sodium-glucose cotransporter-2 inhibitors; GLP-1RA = Glucagon-like peptide-1 receptor agonist

**Table S2.** Subgroup analysis of risk of all stroke between SGLT2i and GLP-1RA groups

| Reference: GLP-1RA | Hazard ratio of SGLT2i | 95% CI | P-value | P-value for interaction |
| --- | --- | --- | --- | --- |
| **Base case** | 1.46 | (0.99,2.17) | 0.058 | NA |
|  |  |  |  |  |
| **Sex** |  |  |  |  |
| Male | 1.88 | (1.15,3.08) | 0.011* | 0.073 |
| Female | 0.89 | (0.45,1.77) | 0.749 |  |
|  |  |  |  |  |
| **Age group** |  |  |  |  |
| <60 years | 1.36 | (0.75,2.47) | 0.308 | 0.918 |
| ≥60 years | 1.55 | (0.91,2.63) | 0.105 |  |
|  |  |  |  |  |
| **Hemoglobin A1c** |  |  |  |  |
| <8% | 2.44 | (1.02,5.88) | 0.046* | 0.393 |
| ≥8% | 1.25 | (0.80,1.95) | 0.321 |  |
|  |  |  |  |  |
| **History of stroke** |  |  |  |  |
| Yes | 3.67 | (1.22,11.02) | 0.020* | 0.043* |
| No | 1.21 | (0.78,1.86) | 0.397 |  |
|  |  |  |  |  |
| **History of atrial fibrillation** |  |  |  |  |
| Yes | 0.38 | (0.04,3.67) | 0.405 | 0.259 |
| No | 1.55 | (1.03,2.32) | 0.034* |  |
|  |  |  |  |  |
| **Duration of diabetes** |  |  |  |  |
| <10 years | 3.73 | (1.58,8.78) | 0.003* | 0.017* |
| ≥10 years | 1.03 | (0.66,1.63) | 0.888 |  |

SGLT2i = Sodium-glucose cotransporter-2 inhibitors; GLP-1RA = Glucagon-like peptide-1 receptor agonist; CI = Confidence interval; NA = Not applicable

Notes:

*Significant at 0.05 level by Cox proportional hazard regression model.

The baseline covariates included in the logistic regression model for propensity score matching were sex, age, hemoglobin A1c, fasting glucose, systolic and diastolic blood pressure, low-density lipoprotein cholesterol, total cholesterol to high-density lipoprotein cholesterol ratio, triglyceride, body mass index, estimated glomerular filtration rate, albuminuria status, duration of diabetes, history of congestive heart failure, hemorrhagic and ischemic stroke, chronic obstructive pulmonary disease, liver disease, severe hypoglycemia, atrial fibrillation, vascular diseases, diabetic retinopathy and diabetic neuropathy, and use of anti-diabetic, anti-hypertensive, lipid-lowering agents, antiplatelets, and anticoagulants.

**Table S3.** Subgroup analysis of risk of ischemic stroke between SGLT2i and GLP-1RA groups

| Reference: GLP-1RA | Hazard ratio of SGLT2i | 95% CI | P-value | P-value for interaction |
| --- | --- | --- | --- | --- |
| **Base case** | 1.53 | (1.01,2.33) | 0.044* | NA |
|  |  |  |  |  |
| **Sex** |  |  |  |  |
| Male | 1.88 | (1.12,3.14) | 0.016* | 0.142 |
| Female | 1.02 | (0.49,2.12) | 0.958 |  |
|  |  |  |  |  |
| **Age group** |  |  |  |  |
| <60 years | 1.28 | (0.69,2.38) | 0.440 | 0.782 |
| ≥60 years | 1.79 | (1.01,3.16) | 0.046* |  |
|  |  |  |  |  |
| **Hemoglobin A1c** |  |  |  |  |
| <8% | 2.28 | (0.94,5.56) | 0.069 | 0.535 |
| ≥8% | 1.34 | (0.84,2.16) | 0.220 |  |
|  |  |  |  |  |
| **History of stroke** |  |  |  |  |
| Yes | 3.66 | (1.22,11.00) | 0.021* | 0.061 |
| No | 1.25 | (0.79,1.99) | 0.338 |  |
|  |  |  |  |  |
| **History of atrial fibrillation** |  |  |  |  |
| Yes | 0.56 | (0.05,6.19) | 0.637 | 0.425 |
| No | 1.59 | (1.04,2.44) | 0.032* |  |
|  |  |  |  |  |
| **Duration of diabetes** |  |  |  |  |
| <10 years | 4.22 | (1.67,10.65) | 0.002* | 0.009* |
| ≥10 years | 1.06 | (0.65,1.71) | 0.821 |  |

SGLT2i = Sodium-glucose cotransporter-2 inhibitors; GLP-1RA = Glucagon-like peptide-1 receptor agonist; CI = Confidence interval; NA = Not applicable

Notes:

*Significant at 0.05 level by Cox proportional hazard regression model.

The baseline covariates included in the logistic regression model for propensity score matching were sex, age, hemoglobin A1c, fasting glucose, systolic and diastolic blood pressure, low-density lipoprotein cholesterol, total cholesterol to high-density lipoprotein cholesterol ratio, triglyceride, body mass index, estimated glomerular filtration rate, albuminuria status, duration of diabetes, history of congestive heart failure, hemorrhagic and ischemic stroke, chronic obstructive pulmonary disease, liver disease, severe hypoglycemia, atrial fibrillation, vascular diseases, diabetic retinopathy and diabetic neuropathy, and use of anti-diabetic, anti-hypertensive, lipid-lowering agents, antiplatelets, and anticoagulants.

**Table S4.** Sensitivity analyses of stroke risk between SGLT2i and GLP-1RA groups

|  | All stroke | | |  | Hemorrhagic stroke | | |  | Ischemic stroke | | |
| --- | --- | --- | --- | --- | --- | --- | --- | --- | --- | --- | --- |
|  | Hazard ratio of SGLT2i (vs. GLP-1RA) | 95% CI | P-value |  | Hazard ratio of SGLT2i (vs. GLP-1RA) | 95% CI | P-value |  | Hazard ratio of SGLT2i (vs. GLP-1RA) | 95% CI | P-value |
| Base case | 1.46 | (0.99,2.17) | 0.058 |  | 1.29 | (0.53,3.14) | 0.582 |  | 1.53 | (1.01,2.33) | 0.044* |
|  |  |  |  |  |  |  |  |  |  |  |  |
| Without censoring on switching treatments | 1.38 | (0.95,1.99) | 0.089 |  | 1.47 | (0.61,3.56) | 0.395 |  | 1.41 | (0.96,2.08) | 0.080 |
| Follow-up duration ≥ 1 year | 1.44 | (0.84,2.47) | 0.186 |  | 0.92 | (0.30,2.79) | 0.882 |  | 1.75 | (0.97,3.17) | 0.062 |
| With at least two dispensing records within 12 months | 1.37 | (0.89,2.13) | 0.157 |  | 1.16 | (0.45,3.00) | 0.753 |  | 1.47 | (0.92,2.36) | 0.108 |
| Initiated treatments on or after 2015 | 1.22 | (0.79,1.88) | 0.364 |  | 0.99 | (0.38,2.57) | 0.981 |  | 1.32 | (0.83,2.10) | 0.237 |
| ‘As-treated’ analysis | 1.60 | (0.92,2.79) | 0.096 |  | 1.23 | (0.36,4.19) | 0.741 |  | 1.77 | (0.97,3.23) | 0.062 |
| Using regression adjustment instead of propensity score matching for adjustment of covariates^†^ | 1.05 | (0.76,1.46) | 0.763 |  | 0.74 | (0.36,1.55) | 0.430 |  | 1.14 | (0.80,1.62) | 0.468 |
| Without any confounding adjustments ^‡^ | 1.28 | (0.94,1.75) | 0.123 |  | 1.09 | (0.55,2.15) | 0.809 |  | 1.34 | (0.96,1.88) | 0.086 |

SGLT2i = Sodium-glucose cotransporter-2 inhibitors; GLP-1RA = Glucagon-like peptide-1 receptor agonist; CI = Confidence interval

Notes:

*Significant at 0.05 level by Cox proportional hazard regression model.

The baseline covariates included in the logistic regression model for propensity score matching were sex, age, hemoglobin A1c, fasting glucose, systolic and diastolic blood pressure, low-density lipoprotein cholesterol, total cholesterol to high-density lipoprotein cholesterol ratio, triglyceride, body mass index, estimated glomerular filtration rate, albuminuria status, duration of diabetes, history of congestive heart failure, hemorrhagic and ischemic stroke, chronic obstructive pulmonary disease, liver disease, severe hypoglycemia, atrial fibrillation, vascular diseases, diabetic retinopathy and diabetic neuropathy, and use of anti-diabetic, anti-hypertensive, lipid-lowering agents, antiplatelets, and anticoagulants.

† Hazard ratios were adjusted by sex, age, hemoglobin A1c, fasting glucose, systolic and diastolic blood pressure, low-density lipoprotein cholesterol, total cholesterol to high-density lipoprotein cholesterol ratio, triglyceride, body mass index, estimated glomerular filtration rate, albuminuria status, duration of diabetes, history of congestive heart failure, hemorrhagic and ischemic stroke, chronic obstructive pulmonary disease, liver disease, severe hypoglycemia, atrial fibrillation, vascular diseases, diabetic retinopathy and diabetic neuropathy, and use of anti-diabetic, anti-hypertensive, lipid-lowering agents, antiplatelets, and anticoagulants, in the Cox proportional hazard regression models.

‡ No adjustment of covariates using either propensity score matching or regression adjustment was performed.
